# Optimization of Dynamic and Buckling Behavior of Thin-Walled Composite Cylinder, Supported by Nature-Inspired Agorithms

**DOI:** 10.3390/ma13235414

**Published:** 2020-11-28

**Authors:** Bartosz Miller, Leonard Ziemiański

**Affiliations:** Faculty of Civil and Environmental Engineering and Architecture, Rzeszów University of Technology, al. Powstańców Warszawy 12, 35-959 Rzeszów, Poland; ziele@prz.edu.pl

**Keywords:** shell, composite, multi-objective optimization, genetic algorithms, artificial neural networks

## Abstract

The paper presents the optimization of stacking sequence (the lamination angles in subsequent composite layers) of the composite cylinder in order to simultaneously maximize the values of the first natural frequency $f_{1}$ and the first buckling force $P_{cr}$. The optimization problem involves either two objective functions or one which combines both problems using a coefficient whose optimal value is also being searched for. The main idea of the paper is the application of two neural network metamodels which substitute very time- and resource-consuming Finite Element (FE) calculations. The metamodels are created separately through a novel iterative procedure, using examples obtained through Finite Element Method (FEM). The metamodels, once ready, are able to assess the values of $f_{1}$ and $P_{cr}$ instantly and thus enable the application of nature-inspired Genetic Algorithm (GA) minimization with reasonable calculation times. Obviously, the maxima of $f_{1}$ and $P_{cr}$ may be located in different points of the design parameters (i.e., lamination angles) space, the considered optimization task is to find a solution for which both $f_{1}$ and $P_{cr}$ simultaneously reach values as close to their maxima as possible. All the investigated optimization examples are repeated several times and basic statistical analysis of the results is presented.

## 1. Introduction

In many engineering fields composite materials are used more and more often, e.g., in aircraft, mechanical, environmental or civil engineering [1,2]. Composites are applied as auxiliary or main structural materials, they have a very desired, high ratio of strength to weight and high durability. Most of the composite cylindrical shells are used under dynamic loading, unfortunately their dynamic behavior have not yet been widely investigated. Understanding this behavior may be crucial in the application of composite materials in structural engineering [3]. Another phenomenon which must be analyzed is buckling [4], associated with a process where a structure suddenly changes its shape. Triggered by a varying external load, this change in configuration often happens in a catastrophic way—named bifurcation buckling—which is predicted by means of an eigenvalue analysis. The composite structures are quite often subjected to in-plane or external loads which may cause buckling.

Multilayer composite structures have a remarkable ease of forming various shapes, while each change of the composite structure topology may significantly change their dynamic behavior and/or buckling properties [5]. In case of layered composite material, created using the same matrix and reinforcement in each of the layers, the properties of the entire composite structure may vary [6,7,8,9]. A change of only the angles of reinforcement placement in subsequent composite material layers may cause the values of a structure natural frequencies and/or buckling forces increase or decrease significantly. Other structural changes, such as a change in the number of composite layers or their thickness, can of course cause substantial changes of a structure properties as well. The possibility of choosing the topological parameters of a composite structure may be very significant from the practical point of view.

Optimization is one of the important stages in the design process. The optimization of static and/or dynamic parameters of a composite structure (e.g., mass, buckling force, stiffness, or the first natural frequency) requires repeatedly calculating the value of the so-called objective function describing the distance of parameters being optimized from their desired values. Real-life engineering problems are typically characterized by more than one objective conflicting with each other. For this reason, an appropriate trade-off between these objective functions should be made using Multi-Objective Optimization (MOO). The computing power demand and time consumption can be reduced if zero-order optimization algorithms are applied (no derivatives of the objective functions are necessary) and modern metamodels of a considered structure are used. The application of nature-inspired metaheuristic algorithms, such as Genetic Algorithms GAs, supported by the use of Neural Networks (NNs) can meet these assumptions [10,11].

Many works have been done on the vibration, buckling and optimization of cylindrical shells. In [8], the author deals with multi-objective optimization of laminated cylindrical shells to maximize a weighted sum of the frequency and buckling load under external load. In [12], multiple objective functions in the optimal design problem of laminated composite plates are considered.

As the design variable the layer fiber orientation may be used, and the multi-objective optimization may also be formulated as the weighted combination of the considered objective functions, dealing e.g., with frequency and buckling force under external load. In [10], a multi-objective optimization strategy for the optimal stacking sequence of laminated cylindrical panels is presented, with respect to the first natural frequency and critical buckling load, using the weighted summation method. Neural networks were used to reproduce the behavior of the structure in both free vibration and buckling conditions, which improved the speed of the optimization process. The paper [13] presents vibration and lateral buckling optimization of thin-walled laminated composite beams with channel sections. While flanges’ width, web’s height, and fiber orientation are simultaneously treated as design variables, the objective function involves maximizing the fundamental natural frequency and critical buckling moment. The problem of optimal stacking sequence for maximization of the natural frequency has also been considered in [14,15,16]. The design of hybrid composite laminates made of high-stiffness skin and low-stiffness core layers was investigated in [17]. The method of simultaneous maximization of fundamental frequency (or the gap between two consecutive frequencies) and minimization of cost by seeking the optimal stacking sequences of both skin and core layers was presented. In the paper [18], a multi-objective design methodology was presented for maximizing the fundamental frequency, buckling load and effective stiffness of laminated composite plates. Lamination parameters were used to characterize the stiffness properties in a compact form and multi-objective optimization solutions was computed in lamination parameter domain for different combinations of design objectives. The multi-objective robust optimisation of T700S carbon/E glass fibre-reinforced epoxy hybrid composites with respect to minimum weight and cost and subject to a prescribed minimum flexural strength has been investigated in [19]. The Pareto optimal front obtained using the NSGA-II and modified hybrid algorithm have been presented and compared.

In this paper the properties of a composite structure are optimized through the changes of the values of basic topological parameters (lamination parameters). The proposed optimization procedure involves nature-inspired optimization algorithms [4] such as GAs [20,21], and Deep Neural Networks (DNNs) [11,22] as a tool to replace time-consuming FEM calculations in dynamic and buckling parameters prediction. The main purpose of this work is to build a multi-objective optimization (for maximizing the first natural frequency and the buckling load) framework for composite circular shells based on lamination parameters. The distributions of Pareto fronts in objective parameter space, which are informative for multi-objective optimization studies, were presented, different materials are also considered. In order to solve the above given optimization task the following problems are considered:

- the application of two separate metamodels for the prediction of two parameters being optimized (the first natural frequency and the buckling load),
- application of an internal feedback (loop) for the metamodels refinement,
- three different approaches to scaling of the objective function arguments,
- full multi-objective approach vs. scalarization approach leading to single-objective approach,
- novel definition of the optimal result of multi-objective optimization problem (Nadir point of the Pareto front).

The analysis of the problems leads finally to a proposition of composite material design by the optimization approach.

## 2. Formulation of the Problem

### 2.1. Solution of the Buckling and Vibration Problems

Once the numerical model of a considered structure is known, the so called initial buckling problem can be described by:

(1) $$\left[K_{L}+\mu K_{\sigma }\left(s^{⋆}\right)\right]v=0,$$

where $K_{L}$ is linear stiffness matrix, $K_{\sigma }\left(s^{⋆}\right)$ is initial stress matrix, μ is a vector of critical load multiplayers (to be determined) and v is a buckling form possible to be obtained once μ is known. The solution of Equation (1) needs prior solution of the pre-buckling state:

(2) $$K_{L}d^{⋆}=P^{⋆},$$

where $P^{⋆}=\mu P$ (for μ=1) is initial load configuration and $d^{⋆}$ is a corresponding displacement state. Once $d^{⋆}$ is known, initial stress matrix $K_{\sigma }\left(s^{⋆}\right)$ can be obtained. Equation (1) can be then solved giving pairs $\left(\mu_{i},v_{i}\right)$, where $\mu_{i}$ is a consecutive critical load multiplayer and $v_{i}$ is a corresponding buckling form. The critical buckling force is given as $P_{cr}=\mu_{1}P$.

The second phenomena analyzed in here is the dynamic behavior of a structure. It can be described by:

(3) $$M\ddot{x}+C\dot{x}+Kx=\bar{P}\;\;,$$

where M, C, and $K=K_{L}$ are mass, damping, and stiffness matrices, respectively, while x and $\bar{P}$ are nodal displacement and external force vectors, respectively. The derivatives with respect to time *t* are marked using dot notation; that is, $\dot{x}=dx/dt$ and $\ddot{x}=d^{2}x/dt^{2}$.

Equation (3), if $\bar{P}\equiv \left(0\right)$ and C≡(0) (i.e., excitation does not occur and the damping is neglected), simplifies to

(4) $$M\ddot{x}+Kx=0.$$

This leads to the generalized eigenproblem [23]:

(5) $$K\Phi =M\Phi \Omega^{2},$$

where the matrix Φ consists of modal shapes $\phi_{i}$ (in columns) and Ω is a diagonal matrix with angular frequencies $\omega_{i}$ matching the vectors $\phi_{i}$. Each of the angular frequencies (also known as radial or circular frequencies, measured in [rad/s]) after dividing by 2π, gives an ordinary frequency (measured in [Hz]):

(6) $$f_{i}=\frac{\omega_{i}}{2\pi },$$

called here the natural frequency $f_{i}$.

### 2.2. Investigated Structure and Its Finite Element Model

The investigated structure is a multilayer composite cylinder. The radius of the cylinder middle surface is R=0.6103 m, the thickness of the shell is t=0.016 m, and the length is l=6.0 m. The considered model was one of the side models investigated in the project carried out at the authors’ university, leading finally to the construction of all-composite road bridge [1]. The walls of the cylinder consist of L=16 composite material layers of equal thickness, where the fiber angles may be different for each layer. The material properties correspond to the graphite-epoxy composite material, the properties of graphite fibers and epoxy matrix are gathered in Table 1. The ratio κ of graphite/epoxy differs from κ=0.1 to κ=0.8, for κ=0.2 the properties of graphite-epoxy composite material are as follows: $E_{1}=62.16$ GPa, $E_{2}=6.23$ GPa, $\nu_{12}=0.42$, $G_{12}=G_{13}=2.40$ GPa, and ρ=1354 kg/m^3^.

**Figure 1:**
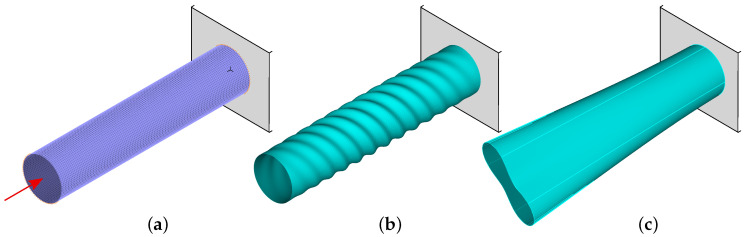
The FE model (**a**) and examples of the first buckling form (**b**) and the first mode shape (**c**).

The finite element model of the investigated structure, shown in Figure 1a, was built using rectangular multi-layered shell 4-node MITC4 elements (first-order shear theory). There were 60 elements in the circumferential direction and 115 elements along the direction of the axis of symmetry, altogether 6900 elements. The overall number of nodes and degrees of freedom was 9680 and 58,080, respectively. The FE model parameters were based on the author’s previous experience, gained while implementing the same model (see [6,24]) and a similar model of thin-walled, composite cylinder. The decision on the finite element mesh and the choice of finite element were made on the basis of detailed studies of FE convergence, the comparison of the results of numerical simulations obtained from two competitive FE codes and the results of experimental tests. The analysis of the dependence of the size of the finite element on the calculated natural frequencies was carried out with the assumption that the obtained accuracy of the numerical model should be at the level determined by the accuracy of the the neural network trained to predict the same values. The neural network accuracy (expressed by the network root mean square error) in the calculations performed by the authors was equal to 0.086 Hz, the adopted FE mesh provided an even lower error of the first natural frequency computations. The results obtained from two FE codes (Adina and Abaqus) were consistent. The experimental tests, involving the measurements of dynamic parameters, were performed on a real scale model of a similar, 9 m long, cantilever thin-walled cylinder, and were carried out at the request of an external company producing composite structures. The tests confirmed that the assumptions made when creating the models of thin-walled cylindrical shells were correct.

The boundary conditions are defined on the shell edges by fixing the translation in all directions (XYZ) at the clamped end of the cylinder. The values of natural frequencies and buckling forces (for the buckling analysis the structure was loaded with an axial force, see Figure 1a) were obtained using the commercial FE code Adina [14].

### 2.3. The GA+DNN Optimization Procedure

The optimization task—for the shell structure made of multi-layer composite material—analyzed here is the simultaneous maximization of the fundamental natural frequency $f_{1}$ and the first buckling force $P_{cr}$. Obviously, the maxima of $f_{1}$ and $P_{cr}$ may be located in different points of the design parameters (i.e., lamination angles) space, the considered optimization task is to find a solution for which both $f_{1}$ and $P_{cr}$ simultaneously reach values as close to their maxima as possible. The Multi-Objective Optimization (MOO) task can be written as

(7) $$\Lambda^{*}=\underset{\Lambda \in L^{L}}{\arg\min}\left\{g^{f}\left(\Lambda \right),g^{P}\left(\Lambda \right)\right\},\;\;\;$$

where $\Lambda =\{\lambda_{1},\lambda_{2},\cdots ,\lambda_{L}\}$ is a vector of L=16 variables (16 lamination angles in consecutive layers of the composite shell), $L^{L}$ is the *L*-dimensional space of the arguments, and $g^{f}\left(\Lambda \right)$ and $g^{P}\left(\Lambda \right)$ are the objective functions to be minimized. The simultaneous maximization of the fundamental natural frequency $f_{1}$ and the first buckling force $P_{cr}$ can also be solved using Scalarization Method (SM) approach [25], where the only scalar objective function $g^{s}\left(\Lambda \right)$ is a linear combination of $g^{f}\left(\Lambda \right)$ and $g^{P}\left(\Lambda \right)$:

(8) $$\Lambda^{*}=\underset{\Lambda \in L^{L}}{\arg\min}\left\{g^{s}\left(\Lambda \right)\right\},\;\;\;$$

(9) $$g^{s}\left(\Lambda \right)=-\left[\alpha \;g^{f}\left(\Lambda \right)+\left(1-\alpha \right)\;g^{P}\left(\Lambda \right)\right],\;\mathrm{for}\;0\leq \alpha \leq 1.$$

where α is a weighting factor. Both approaches are applied in what follows.

The objective functions $g^{f}\left(\Lambda \right)$ and $g^{P}\left(\Lambda \right)$ are defined using the following formulas:

(10) $$g^{f}\left(\Lambda \right)=-\frac{f_{1}\left(\Lambda \right)}{f_{1}^{0}}$$

(11) $$g^{P}\left(\Lambda \right)=-\frac{P_{cr}\left(\Lambda \right)}{P_{cr}^{0}}$$

where $f_{1}^{0}$ and $P_{cr}^{0}$ are the scaling factors.

In order to make the optimization procedure less time-consuming, the value of the first natural frequency for the given lamination angles (as gathered in vector Λ) was calculated—instead of the usual FEM calculations—using Deep Neural Network (DNN) based *metamodel*: $f_{1}={\mathrm{DNN}}^{f_{1}}\left(\Lambda \right)$, the value of the first buckling force $P_{cr}$ was calculated using separate neural network metamodel: $P_{cr}={\mathrm{DNN}}^{P_{cr}}\left(\Lambda \right)$. Finally, Genetic Algorithm (GA) was applied to solve either the MOO or SM optimization problem (i.e., to find the lamination angles that yield the maximum value of the fundamental natural frequency $f_{1}$ and simultaneously the maximum value of the first buckling force $P_{cr}$). Non-dominated Sorting Genetic Algorithm of type II (NSGA-II) has been applied, which has the following main features: “uses an elitist principle, uses an explicit diversity preserving mechanism, emphasizes non-dominated solutions” [26] and low computational requirements [27]. This algorithm is considered to be one of the standard approaches and is therefore implemented in many math codes [28,29].

The above described approach—involving two metamodels—differs from usual nature-inspired approach to multi-objective optimization, where one metamodel is applied. Since $f_{1}$ and $P_{cr}$ are predicted using the same input data (lamination angles gathered in vector Λ) such a metamodel may predict both $f_{1}$ and $P_{cr}$ simultaneously:

(12) $$\left[f_{1},P_{cr}\right]=DNN^{f_{1},P_{cr}}\left(\Lambda \right).$$

**Figure 2:**
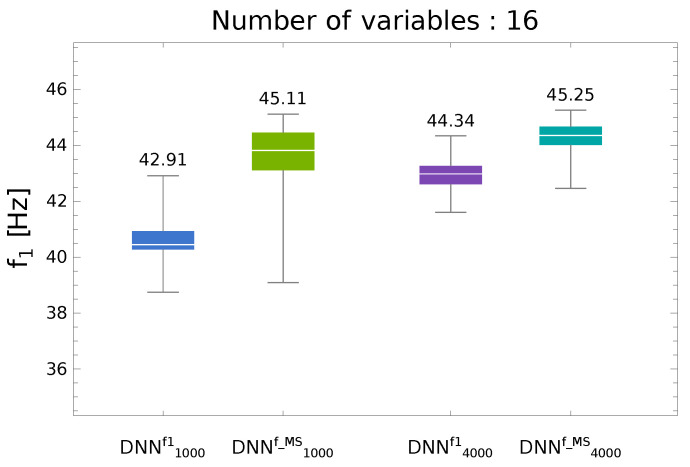
The accuracy of $f_{1}$ maximization using either $f_{MS}={\mathrm{DNN}}^{f_{1}}\left(\Lambda \right)$ or $f_{MS}={\mathrm{DNN}}^{f_{MS}}\left(\Lambda \right)$ metamodels.

As it was shown by Miller and Ziemiański in [30] for a graphite-epoxy composite material with graphite/epoxy ratio κ=0.2, a significantly higher accuracy of $f_{1}$ prediction by DNN metamodel and in consequence better results of $f_{1}$ maximization are obtained when not the set of first natural frequencies (even the one containing only $f_{1}$) is predicted but when the DNN metmodel predicts a set of natural frequencies $f_{MS}$ matching selected mode shapes gathered in a M set, namely $f_{MS}\;=\;{\mathrm{DNN}}^{f_{MS}}\left(\Lambda \right)$ (see Figure 2). The learning data for ${\mathrm{DNN}}^{f_{MS}}$ metamodel have to be pre-processed, namely the mode shapes obtained for a given Λ have to be identified and the natural frequencies are arranged according to the mode shapes they correspond to, instead of usually applied ascending order. If the considered mode shapes are gathered in M set and the corresponding natural frequencies are gathered in $f_{MS}$ the mode shape-based metamodel is given as:

(13) $$f_{MS}={\mathrm{DNN}}^{f_{MS}}\left(\Lambda \right).$$

**Figure 3:**
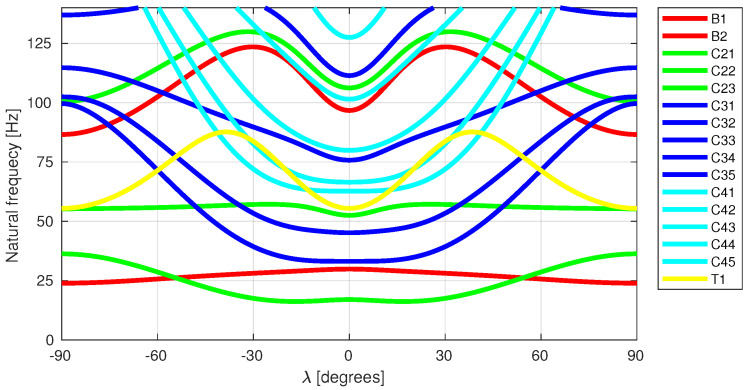
Natural frequencies variations in 3-layer cylinder, stacking sequence [λ/0/λ].

Higher accuracy of the metamodel defined by Equation (13) is related to the fact that the first natural frequency $f_{1}$ corresponds—for different lamination angles—to different mode shapes (see Figure 3). The ${\mathrm{DNN}}^{f_{1}}\left(\Lambda \right)$ metamodel is therefore less precise than ${\mathrm{DNN}}^{f_{MS}}\left(\Lambda \right)$.

The diagram presented in Figure 2 is a boxplot representing the following values describing the analyzed population of results (250 repetitions of optimization process were performed): the maximum (including possible outliers), the third quartile (Q75%, the upper limit of the box), the median, the first quartile (Q25%, the lower limit of the box), and the minimum (including possible outliers). The data presented in Figure 2 were obtained for a structure the same as the one analyzed in this paper but with different material properties (see [30]). The superscript ${\left(\cdot \right)}^{f_{1}}$ or ${\left(\cdot \right)}^{f_{MS}}$ in metamodel names informs whether the metamodel predicted directly $f_{1}$ or a set of frequencies $f_{MS}$. The subscript ${\left(\cdot \right)}_{1000}$ and ${\left(\cdot \right)}_{4000}$ in metamodel names shows the number of patterns at the disposal during the metamodel creation phase. In what follows ${\mathrm{DNN}}_{n000}^{f_{MS}}\left(\Lambda \right)$ will be called Pn (*n* stands for the number of patterns divided by 1000).

The vector $f_{MS}$ is composed as a set of natural frequencies matching eleven selected mode shapes (three circumferential modes with the second circumferential wave: C21, C22, C23; three circumferential modes with the third circumferential wave: C31, C32, C33; two circumferential modes with the fourth circumferential wave: C41, C42; two bending modes: B1, B2; and one torsional mode: T1):

(14) $$f_{MS}=\left\{f_{C21},f_{C22},f_{C23},f_{C31},f_{C32},f_{C33},f_{C41},f_{C42},f_{B1},f_{B2},f_{T1}\right\}.$$

The number of the mode shapes selected to create the $f_{MS}$ vector was determined by the arbitrarily selected limiting value of 100 Hz; thus, the vector should include all natural frequencies smaller than this limit value.

The first buckling force $P_{cr}$ matches always—in the analyzed range—the same buckling shape, so it was not necessary to apply for $P_{cr}$ prediction a similar approach as in the case of $f_{1}$ prediction. Moreover, the application of one metamodel

(15) $$\left[f_{MS},P_{cr}\right]={\mathrm{DNN}}^{f_{MS},P_{cr}}\left(\Lambda \right)$$

gives worse accuracy than the application of two separate metamodels.

In all the above discussed cases instead of deep networks other data-driven models could be applied, DNNs were chosen since they work very well with huge learning sets and the description of 16-dimensional space of lamination angles needs at least a few thousands examples.

The decision of applying the vector $f_{MS}$, composed of natural frequencies matching selected mode shapes, leads to a new definition of the objective function $g^{f}\left(\Lambda \right)$:

(16) $$g^{f}\left(\Lambda \right)=-\frac{\min\;f_{MS}\left(\Lambda \right)}{f_{1}^{0}},\;\mathrm{for}\;MS\in M.$$

The objective function definition requires prior identification of the mode shapes and corresponds to the maximization of the lowest of the natural frequencies matching the selected mode shapes.

The main optimization (either MOO or SM) is preceded by the creation of two DNN metamodels, which predicted the model natural frequencies (the ${\mathrm{DNN}}^{f_{MS}}$ metamodel) and the first buckling force (the ${\mathrm{DNN}}^{P_{cr}}$ metamodel) for the given set of model parameters. The metamodels are Deep Neural Networks (DNN), trained on examples generated using FE model, which approximate the values of $f_{MS}$ or $P_{cr}$, respectively. The accuracy of metamodel approximation is verified through the single-objective maximization of $f_{1}$:

(17) $$\Lambda^{*}=\underset{\Lambda \in L^{L}}{\arg\min}\left\{g^{f}\left(\Lambda \right)\right\}=\underset{\Lambda \in L^{L}}{\arg\min}\left\{-\frac{\min\;f_{MS}^{⋆}\left(\Lambda \right)}{f_{1}^{0}}\right\},\;\mathrm{for}\;MS\in M,$$

or $P_{cr}$:

(18) $$\Lambda^{*}=\underset{\Lambda \in L^{L}}{\arg\min}\left\{g^{P}\left(\Lambda \right)\right\}=\underset{\Lambda \in L^{L}}{\arg\min}\left\{-\frac{P_{cr}^{⋆}\left(\Lambda \right)}{P_{cr}^{0}}\right\},$$

where the star ${\left(\cdot \right)}^{⋆}$ in $f_{MS}^{⋆}$ and $P_{cr}^{⋆}$ means that those values were obtained from the appropriate metamodels.

**Figure 4:**
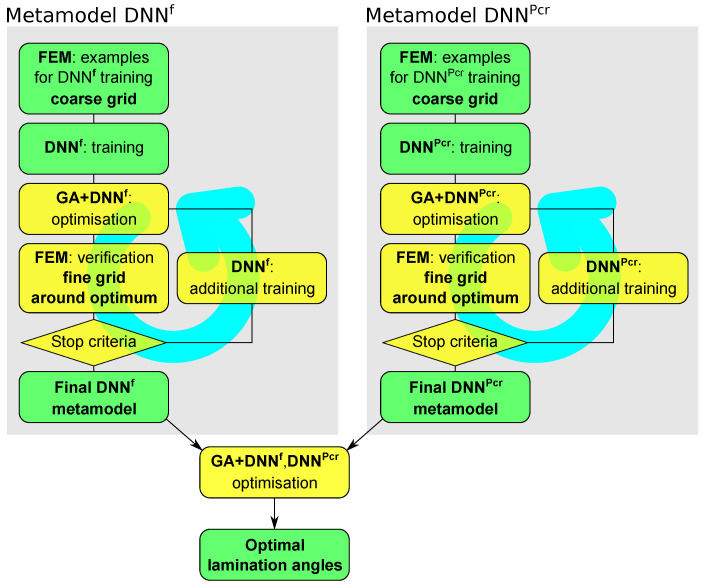
The applied optimization procedure, metamodels creation procedure with the *Curriculum Learning* loop, see [24,30].

The whole optimization procedure (either MOO or SM), together with the metamodels creation phase, is presented in Figure 4.

In order to provide a clear description of the metamodels creation procedure a new symbol of o is introduced. In case of metamodel predicting the natural frequencies $o=f_{MS}$ and $g^{o}\left(\Lambda \right)=g^{f}\left(\Lambda \right)$, in case of metamodel predicting the buckling force $o=\{P_{cr}\}$ and $g^{o}\left(\Lambda \right)=g^{P_{cr}}\left(\Lambda \right)$.

The idea, proposed by Miller and Ziemiański in [24] for maximization of $f_{1}$ and now adopted to the problem of simultaneous maximization of $f_{1}$ and $P_{cr}$, consists of the following steps, performed separately for both metamodels:

1. FEM examples for DNN^**o**^ training (coarse grid): $L^{n}$ space is intentionally described by a limited number of examples (so called *patterns*) $P^{o}={\left\{{\left(o,\Lambda \right)}^{j}\right\}}_{j=1}^{P}$, where o is obtained using FE calculations: o=FE(Λ). The role of the patterns is not to precisely describe the value of the objective function $g^{o}\left(\Lambda \right)$ but to approximately locate the extremes of this function.
2. DNN^**o**^ training: A deep network is trained to map Λ into o; that is, DNN^**o**^ should be capable of working as function that returns vector $o^{j}$ for a given vector $\Lambda^{j}$: $o^{j}={\mathrm{DNN}}^{o}\left(\Lambda^{j}\right)$.
3. GA+DNN^**o**^ optimization: single-objective GA optimization (maximization of o), with DNN^**o**^ applied to calculate the objective function, is performed; the result of this optimization is not significant with regard to a single $\Lambda^{*}$ vector; the number of repetitions of this procedure should produce a set of $\Lambda^{*,k}$ vectors around the global minimum. Since there is a usual system shift of the results of GA+DNN^**o**^ optimization relative to the results of FEM (herein called “real” results), it would be advisable to interrupt GA optimization before it reaches any sharp minimum of DNN^**o**^ approximation of the objective function (possibly shifting from the “real” minimum of the objective function being sought).
4. FEM verification (fine grid around the optimum): For each $\Lambda^{*,k}$ obtained in the previous step (there is not a single vector $\Lambda^{*,k}$ but a group of vectors since the tentative optimization in the previous step is repeated *K* times), a vector of parameters being predicted $o^{*,k}$ is calculated $o^{*,k}=\mathrm{FEM}\left(\Lambda^{*,k}\right)$, and a new set of patterns is created $P^{o,*}={\left\{{\left(o^{*},\Lambda^{*}\right)}^{k}\right\}}_{k=1}^{K}$.
5. Stop criteria: The usual stop criteria are verified; if the criteria are not fulfilled, the procedure returns to GA+DNN^**o**^ optimization following additional DNN^**o**^ training (retraining).
6. DNN^**o**^ additional training: The DNN^**o**^ already trained in the second step of the procedure is trained again (retrained) with the original set of patterns $P^{o}$ expanded with $P^{o,*}$; the DNN^**o**^ should then be more precise in the area of the expected global minimum.

The iterative searching for a precise metamodel, described above in Point 6 is called Curriculum Learning (CL). The number of CL loops applied is denoted by *i* in CL*i* (e.g., CL**1** means that one CL loop is applied). Since two metamodels are applied, it is possible to refine them separately.

Once both DNN^**o**^ metamodels (DNN${}^{f_{MS}}$ and DNN${}^{P_{cr}}$) are ready, the main optimization task (MOO or SM) is performed without further application of time-consuming FE model. The application of the numerical model (or, when possible, analytical one) is necessary only to tune the metamodels and to verify the obtained results.

All DNN training and testing processes were performed in the Mathematica (V12.0, Wolfram Research Inc., Champaign, IL, USA) environment [28], the completed ANNs were then transferred to Matlab (R2019a, The MathWorks Inc., Natick, MA, USA) [29], where GA optimization phase was conducted.

## 3. Optimization of the Cantilever Cylinder

### 3.1. Metamodel ${\mathrm{DNN}}^{f_{MS}}$ and Single-Objective Optimization of $f_{1}$

**Figure 5:**
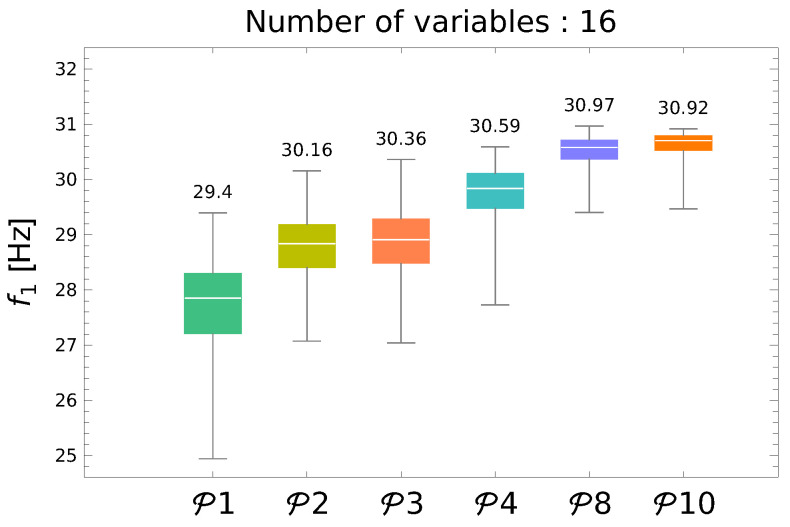
Maximization of $f_{1}$ through GA-DNN procedure.

According to the procedure shown in Figure 4 the creation of ${\mathrm{DNN}}^{f_{MS}}\left(\Lambda \right)$ starts with the generation of a set of patterns describing the relation between randomly chosen parameter vectors Λ and selected natural frequencies gathered in $f_{MS}$, where natural frequencies were computed using FEM: $f_{MS}=\mathrm{FEM}\left(\Lambda \right)$. Deep networks were then trained to mimic this relation, and so the neural metamodel was obtained:

(19) $$f_{MS}\approx {\mathrm{DNN}}^{f_{MS}}\left(\Lambda \right)$$

and the GA-DNN optimization was performed with $g^{f_{1}}\left(\Lambda \right)$ objective function (see Equation (17)). The summary of $f_{1}$ maximization results is shown in Figure 5 (for the details of boxplots see the description of Figure 2).

Each of boxes in Figure 5 shows the results of 250 repetitions of GA-DNN${}^{f_{MS}}$ optimization with the metamodel trained using Pn set of patterns, where *n* shows the number of patterns divided by 1000. The improvement of the maximization results with the increase of the number of employed metamodel training patterns is clearly visible; however, over 8000 of patterns the increase disappears. For the main task—the simultaneous maximization of $f_{1}$ and $P_{cr}$—the metamodel trained using P8 set of patterns (called here ${\mathrm{DNN}}_{8000}^{f_{MS}}$) has been applied.

The maximal value of $f_{1}$ obtained from ${\mathrm{DNN}}_{8000}^{f_{MS}}$, namely 30.97 Hz, is in what fallows called $f_{1}^{max}$. It is used as one of the scaling factors $f_{1}^{0}$ in the main optimization procedure.

### 3.2. Metamodel ${\mathrm{DNN}}^{P_{cr}}$ and Single-Objective Optimization of $P_{cr}$

**Figure 6:**
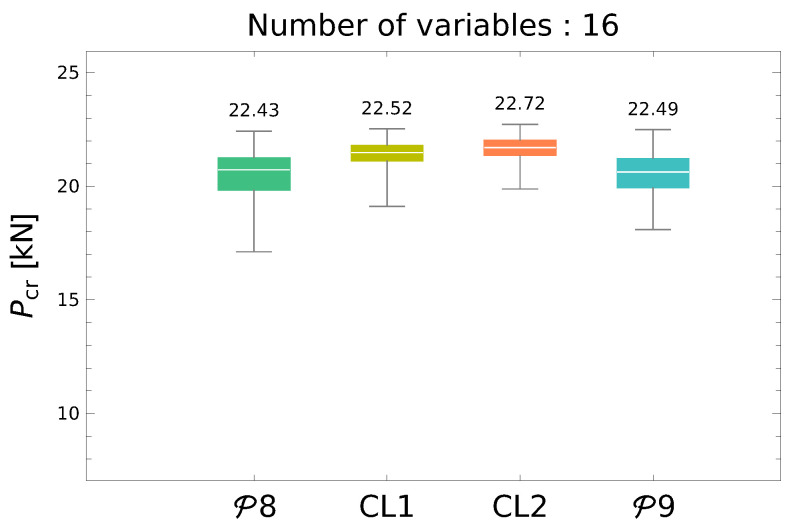
Maximization of $P_{cr}$ through GA-DNN procedure.

In the second initial step to the main optimization procedure the metamodel ${\mathrm{DNN}}^{P_{cr}}$ was created and verified during a single-objective maximization of $P_{cr}$. A number of new example sets, composed of different number of randomly generated vectors Λ and corresponding FEM-generated critical forces $P_{cr}=\mathrm{FEM}\left(\Lambda \right)$, were tried out. The appropriate pattern sets were generated, according to the scheme in Figure 4, independently of the patterns to be used for ${\mathrm{DNN}}^{f_{MS}}$ metamodel training. In this case, the increase in the number of patterns did not cause a satisfactory increase in metamodel accuracy, so the *Curriculum Learning* loop—presented in Figure 4—was applied. In each of the analyzed cases CL loops caused significant improvement of the metamodel accuracy and $P_{cr}$ maximization results, an example of this phenomenon for ${\mathrm{DNN}}_{8000}^{P_{cr}}$ metamodel is presented in Figure 6.

For the main task, the simultaneous maximization of $f_{1}$ and $P_{cr}$, the P8-CL2 metamodel (also called ${\mathrm{DNN}}_{8000}^{P_{cr}}\text{-}\mathrm{CL}2$) has been applied.

The maximal value of $P_{cr}$ obtained from ${\mathrm{DNN}}_{8000}^{P_{cr}}\text{-}\mathrm{CL}2$, namely 22.72 Hz, is in what fallows called $P_{cr}^{max}$. It is used as one of the scaling factors $P_{cr}^{0}$ in the main optimization procedure.

### 3.3. Multi-Objective Optimization of $f_{1}$ and $P_{cr}$

The metamodels ${\mathrm{DNN}}_{8000}^{f_{MS}}$ and ${\mathrm{DNN}}_{8000}^{P_{cr}}\text{-}\mathrm{CL}2$ were applied as tools to predict the values of parameters being minimized within a GA optimization procedure with the objective function given by Equation (7). After initial test the main GA parameters were selected as follows: floating-point coding of parameters, 100 individuals in each population, 500 generations, tournament selection function, adaptive feasible mutation function, constraints $-90\leq \lambda_{i}\leq 90$ applied on each lamination angle $\lambda_{i}$ (for details of Matlab implementation of GA see [29]). The optimization procedure was repeated 250 times in order to get a statistical description of the obtained results, moreover each result (final $f_{1}^{⋆}$ and $P_{cr}^{⋆}$ values obtained by metamodels for a particular lamination angles vector Λ) was verified using FEM.

Three different variants of scaling factors pairs (see Equations (11) and (16)) have been considered:

(a) no scaling at all: $f_{1}^{0}=P_{cr}^{0}=1$,
(b) scaling to an arbitrarily selected lamination angles case: $f_{1}^{0}$ and $P_{cr}^{0}$ obtained for $\Lambda ={\left[45/-45\right]}_{8}$ [10],
(c) scaling to maximal values of $f_{1}$ and $P_{cr}$ obtained in previous steps: $f_{1}^{0}=30.97$ Hz and $P_{cr}^{0}\;=\;22.72$ MN.

As a result of each of the above described 250 repetitions of MOO a cloud of points is obtained, each of them being an element of one of 250 so called Pareto Fronts (PFs) obtained in each of the repetitions. Pareto front is a border line between the feasible and the infeasible results, given in the optimized parameters’ space, here in $\left(f_{1},P_{cr}\right)$ two-dimensional space. In other words, the PF gathers all non-dominated results, in the sense that for the points on PF there is not possible to improve any of the $\left(f_{1},P_{cr}\right)$ values without worsening the other.

**Figure 7:**
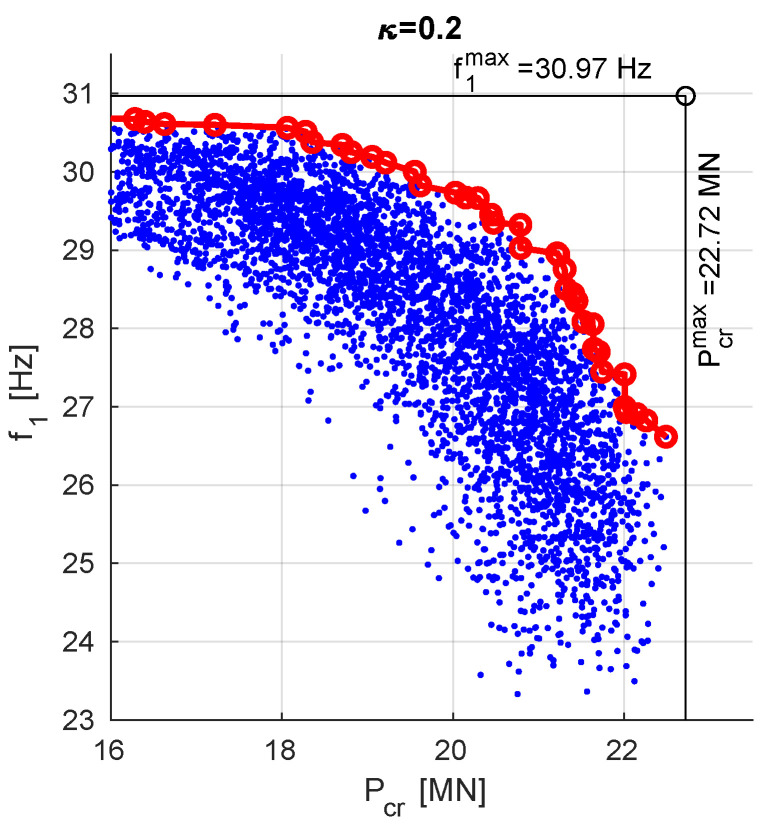
Pareto front obtained through MOO procedure, $f_{1}^{0}=P_{cr}^{0}=1$.

Figure 7 shows the cloud of points (in blue) obtained from MOO involving GA and DNN metamodels and verified through FEM, namely for each of the points $\left(f_{1}^{⋆},P_{cr}^{⋆}\right)$ obtained from MOO involving neural metamodels, FE verification was carried out and a number of $\left(f_{1}^{FE},P_{cr}^{FE}\right)$ points was obtained. Figure 7 shows also the values of $f_{1}^{max}$ and $P_{cr}^{max}$ obtained during the initial phase of neural metamodels creation and so called Utopia Point (UP, see [25,31]) at their intersection. The scaling factors $f_{1}^{0}$ and $P_{cr}^{0}$ applied in this approach were both equal to 1 (so no scaling was performed).

Although the points in Figure 7 are all the elements of PFs obtained in independent repetitions of MOO, the final PF is obtained from the analysis of all the partial results. The red line in Figure 7 connects all the non-dominated results.

**Figure 8:**
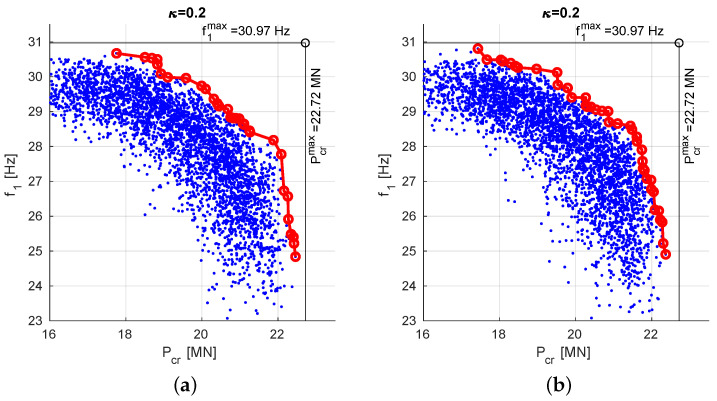
Pareto front obtained through MOO procedure: (**a**) $f_{1}^{0}$ and $P_{cr}^{0}$ obtained for $\Lambda ={\left[45/-45\right]}_{8}$, (**b**) $f_{1}^{0}=f_{1}^{max}=30.97$ Hz; $P_{cr}^{0}=P_{cr}^{max}=22.72$ MN.

Figure 8 shows final PFs obtained for the other considered values of scaling factors, namely:

(a) scaling to an arbitrarily selected lamination angles case: $f_{1}^{0}$ and $P_{cr}^{0}$ obtained for $\Lambda ={\left[45/-45\right]}_{8}$,
(b) scaling to maximal values of $f_{1}$ and $P_{cr}$ obtained in previous steps: $f_{1}^{0}=30.97$ Hz and $P_{cr}^{0}\;=\;22.72$ MN.

Table 2 shows the data describing the so called Nadir Point (NP, see [31]). Usually NP is selected as the one giving the minimal Euclidean distance $D_{NU}$, from Utopia Point (UP) whose coordinates are $\left(f_{1}^{max},P_{cr}^{max}\right)$:

(20) $$D_{NU}=\min\sqrt{{\left(\frac{f_{1}^{utopia}-f_{1}}{f_{1}^{norm}}\right)}^{2}+{\left(\frac{P_{cr}^{utopia}-P_{cr}}{P_{cr}^{norm}}\right)}^{2}}=\min\sqrt{{\left(\frac{f_{1}^{max}-f_{1}}{f_{1}^{max}}\right)}^{2}+{\left(\frac{P_{cr}^{max}-P_{cr}}{P_{cr}^{max}}\right)}^{2}}$$

Here the normalization factors in the objective space are $f_{1}^{utopia}=f_{1}^{norm}=f_{1}^{max}$ and $P_{cr}^{utopia}=P_{cr}^{norm}=P_{cr}^{max}$:

This approach to the selection of NP prefers one or the other of the minimized values (depending on the chosen scaling variant), another approach is thus proposed. Table 3 shows the NPs coordinates, where NP is the point number $i^{*}$ giving the maximum of the minimal value among $f_{1}/f_{1}^{max}$ and $P_{cr}/P_{cr}^{max}$:

(21) $$i^{*}=\underset{i}{\arg\max}\left(\min\left(\frac{f_{1}^{i}}{f_{1}^{max}},\frac{P_{cr}^{i}}{P_{cr}^{max}}\right)\right)$$

The analysis of Figure 7 and Figure 8 and Table 2 and Table 3 reveals that there are are no significant qualitative differences between three scaling approaches.

### 3.4. Scalarization Method Approach to Optimization of $f_{1}$ and $P_{cr}$

The same metamodels (${\mathrm{DNN}}_{8000}^{f_{MS}}$ and ${\mathrm{DNN}}_{8000}^{P_{cr}}\text{-}\mathrm{CL}2$) together with GA optimization, were applied as a tool to solve the SM approach to the optimization problem, with the objective function given by Equation (9). After initial test the main GA parameters were selected as follows: floating-point coding of parameters, 50 individuals in each population, 100 generations, stochastic uniform selection function, Gaussian mutation function, constraints $-90\leq \lambda_{i}\leq 90$ applied on each lamination angle $\lambda_{i}$ (for details of Matlab implementation of GA see [29]). The optimization procedure was repeated 250 times in order to get a statistical description of the obtained results, moreover each result (final $f_{1}$ and $P_{cr}$ values obtained by metamodels for a particular lamination angles vector Λ) was verified using FEM.

**Figure 9:**
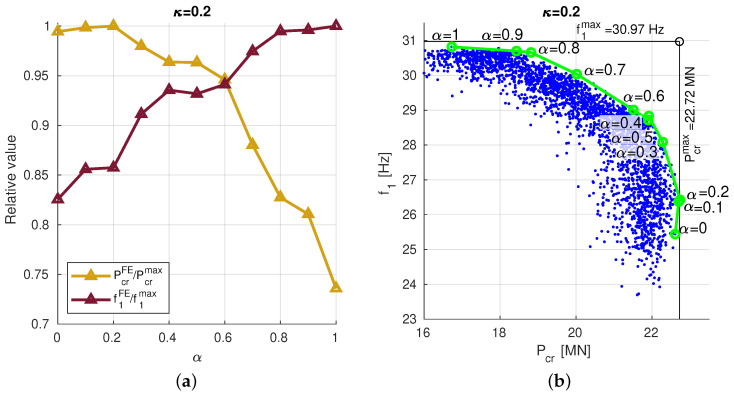
The results of $f_{1}$ and $P_{cr}$ optimization, obtained for different values of coefficient α and constant κ=0.2, $f_{1}^{0}=P_{cr}^{0}=1$: (**a**) only the best results for each κ, (**b**) all the obtained results, presented in two-dimensional coordinate system of the results ($P_{cr}$-$f_{1}$).

The results, obtained for scaling factors $f_{1}^{0}$ and $P_{cr}^{0}$ computed for the lamination angle case $\Lambda ={\left[45/-45\right]}_{8}$, for graphite/epoxy ratio κ=0.2, are presented in Figure 9a.

The best values of $f_{1}$ and $P_{cr}$, obtained from the SM optimization procedure for different α coefficient values (0≤α≤1) and are—only to create a plot—divided by $f_{1}^{max}=30.97$ Hz or $P_{cr}^{max}=22.72$ MN, respectively. The intersection of the two lines ($f_{1}/f_{1}^{max}$ and $P_{cr}/P_{cr}^{max}$) in Figure 9a shows the location of NP fulfilling the condition given in the previous section: the maximum of the minimal value among $f_{1}/f_{1}^{max}$ and $P_{cr}/P_{cr}^{max}$. For the SM optimization procedure such a result is obtained for α≈0.6 and reads $f_{1}=29.1$ Hz (94% of $f_{1}^{max}$) and $P_{cr}=21.3$ MN (93.9% of $P_{cr}^{max}$).

**Figure 10:**
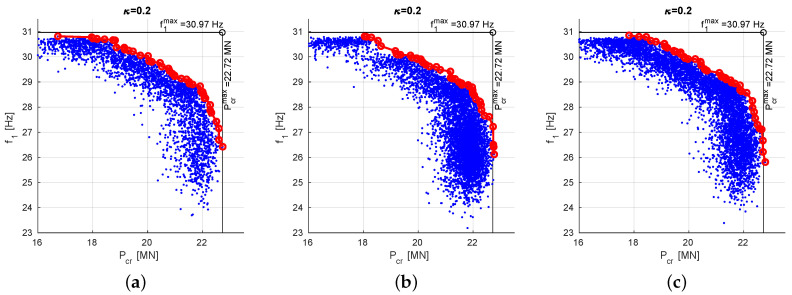
The envelope Pareto front, obtained for different values of coefficient α and constant κ=0.2, (**a**) $f_{1}^{0}=P_{cr}^{0}=1$, (**b**) $f_{1}^{0}$ and $P_{cr}^{0}$ obtained for $\Lambda ={\left[45/-45\right]}_{8}$, (**c**) $f_{1}^{0}=f_{1}^{max}$, $P_{cr}^{0}=P_{cr}^{max}$.

The same results are shown (in green) also in Figure 9b, the only difference is that they are presented in the two-dimensional coordinate system of the results ($P_{cr}$-$f_{1}$). The blue points in this figure represent the dominated solutions among all the other results obtained from SM optimization procedures (250 repetitions for each considered value of α). The green line, connecting the best results obtained for different values of α, may be considered as a kind of PF. In order to make the plots compatible with the plots created for MOO also the PF is created as the line connecting all the non-dominated results and presented (in red) in Figure 10 for different scaling cases. The optimal results (Nadir points) are gathered in Table 4, NPs are obtained using the same approach as in Table 3 and show slightly better accuracy of SM approach.

### 3.5. Multi-Objective Optimization vs. Scalarization Method

**Figure 11:**
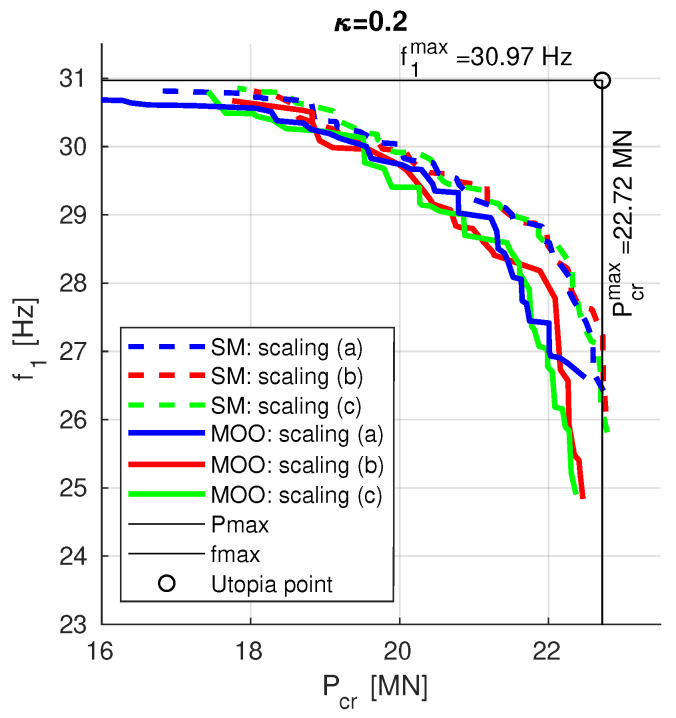
The final PFs obtained from MOO (continuous lines) and SM (dashed lines) optimization.

The results obtained from MOO and SM optimization are gathered in Figure 11. Each line presents the Pareto front obtained either from MOO (continuous line) of from SM optimization (dashed line), moreover three different variants of scaling factors pairs have been considered: (a) no scaling at all: $f_{1}^{0}=P_{cr}^{0}=1$, (b) scaling to an arbitrarily selected lamination angles case: $f_{1}^{0}$ and $P_{cr}^{0}$ obtained for $\Lambda ={\left[45/-45\right]}_{8}$, (c) scaling to maximal values of $f_{1}$ and $P_{cr}$ obtained in previous steps: $f_{1}^{0}=30.97$ Hz and $P_{cr}^{0}=22.72$ MN.

The differences between scaling scenarios are negligible, there is however subtle but clear advantage of SM over MOO approach.

## 4. Simultaneous Maximization of $f_{1}$ and $P_{\mathrm{cr}}$ for Varying Graphite/Epoxy Ratio

**Figure 12:**
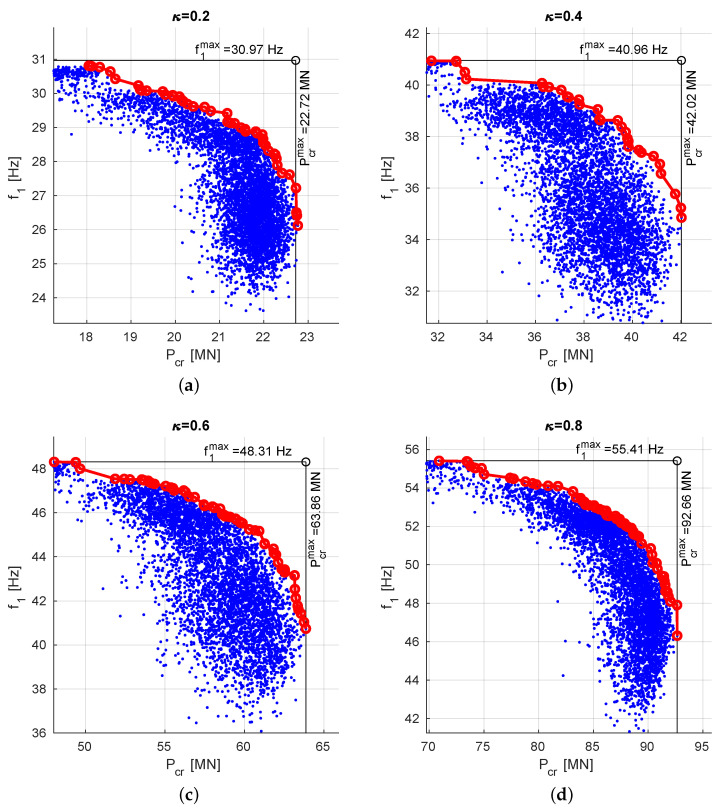
The results of $f_{1}$ and $P_{cr}$ optimization, obtained for different values of coefficient κ, (**a**) κ=0.2, (**b**) κ=0.4, (**c**) κ=0.6, (**d**) κ=0.8.

The above described maximization of $f_{1}$ and $P_{cr}$ was performed for different fiber/matrix ratio κ. Only SM approach was applied, with $f_{1}^{0}$ and $P_{cr}^{0}$ obtained for $\Lambda ={\left[45/-45\right]}_{8}$ (scaling to an arbitrarily selected lamination angles case). The results are shown in Figure 12 and in Table 5.

The results gathered in Table 4 and Table 5 show that using the SM approach it is possible to obtain the optimal results with both $f_{1}$ and $P_{cr}$ only 6% smaller than their maximum values.

**Figure 13:**
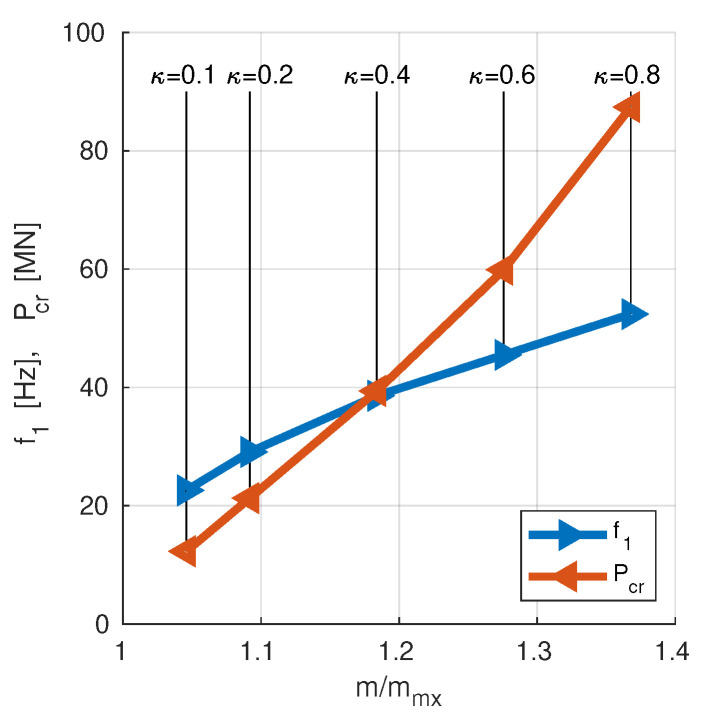
The optimal values of $f_{1}$ and $P_{cr}$ in relation of the relative mass of the cylinder.

Figure 13 show the dependence of NP coordinates (optimal values of $f_{1}$ and $P_{cr}$) on κ (ratio of graphite fibers to epoxy matrix), and thus on the mass *m* of the cylinder. The horizontal axis of Figure 13 shows the relative mass of the investigated cylinder where *m* is an overall mass and $m_{mx}$ is a theoretical mass of the cylinder made of the epoxy matrix only, without any graphite fibers (κ=0). An additional case with κ=0.1 is included.

The analysis of Figure 13 allows to design a composite material for the analyzed cylinder to obtain the expected properties of the investigated structure.

## 5. Conclusions

The paper presents the optimization of stacking sequence (the lamination angles in subsequent composite layers) of the composite cylinder in order to maximize simultaneously values of the first natural frequency $f_{1}$ and the first buckling force $P_{cr}$. The optimization problem is solved using:

- two separate metamodels,
- CL loops for metamodels refinement,
- multi-objective optimization with two objective functions, or
- scalarization method approach, where the only scalar objective function is a linear combination of two objective functions involved in the previous approach.

Moreover, three different scaling of the input data for the optimization procedure are verified:

- no scaling at all: $f_{1}^{0}=P_{cr}^{0}=1$,
- scaling to an arbitrarily selected lamination angles case: $f_{1}^{0}$ and $P_{cr}^{0}$ obtained for $\Lambda ={\left[45/-45\right]}_{8}$,
- scaling to maximal values of $f_{1}^{0}=f_{1}^{max}$ and $P_{cr}^{0}=P_{cr}^{max}$ obtained during metamodel creation phase.

New proposition of ND (optimal result) selection is also proposed.

In the presented examples the scalarization method gives slightly better results, while the three investigated scaling approaches are barely distinguishable.

The two neural network metamodels substitute very time- and resource-consuming FE calculations. The metamodels are created using examples obtained through FEM, but once the metamodels are ready they are able to assess the values of $f_{1}$ and $P_{cr}$ instantly and thus enable the application of nature-inspired GA minimization with no further involvement of time-consuming FEM. The application of the proposed approach reduces the number of necessary FE calls by about two orders of magnitude (from 2,500,000 to 26,500) what gives huge time and resource consumption savings in each of the considered cases.

The applied metamodels enable the precise tuning of the investigated structure parameters, it is possible to obtain such a values of the design parameters (i.e., the lamination angles of laminate layers) that the value of the fundamental natural frequency reaches a value close to its maximum, simultaneously with the buckling force also being near its maximum. In fact in every considered case the final solution gives the values of both $f_{1}$ and $P_{cr}$ smaller then the maximum values by only 6%.

Genetic algorithms and DNN are very suitable tools to find global (or near-global) optimal solution in the analyzed problems, where laminated composite is used.

The presented approach allows to design cylinder composite material through optimization approach.

The research should be carried out further, the following problems should be addressed:

- other parameters—like overall mass and/or stiffness—should be taken into account,
- wider range of control variables, also some geometric and/or material properties should be considered,
- CL approach on the level of the whole MOO procedure should be applied, not only on the level of metamodel creation.

## Figures and Tables

**Table 1:** Material properties of graphite fibers and epoxy matrix.

	E (GPa)	ν (-)	ρ (kg/m^3^)
graphite fibers	294.0	0.2	1810
epoxy matric	62.16	0.3	1240

**Table 2:** Nadir points selected as the ones giving minimal Euclidean distance to UP, norm=max.

Scaling Factors	$f_{1}$ Hz	$f_{1}/f_{1}^{\max}$ %	$P_{\mathrm{cr}}$ MN	$P_{\mathrm{cr}}/P_{\mathrm{cr}}^{\max}$ %
$f_{1}^{0}=1$, $P_{cr}^{0}=1$	30.4	99.0%	18.8	82.8%
$f_{1}^{0}$ and $P_{cr}^{0}$ obtained for $\Lambda ={\left[45/-45\right]}_{8}$	30.8	99.4%	18.3	80.5%
$f_{1}^{0}=f_{1}^{max}$, $P_{cr}^{0}=P_{cr}^{max}$	30.5	98.6%	19.1	84.1%

**Table 3:** Nadir points selected as the ones maximizing the relative values of $f_{1}$ and $P_{cr}$.

Scaling Factors	$f_{1}$ Hz	$f_{1}/f_{1}^{\max}$ %	$P_{\mathrm{cr}}$ MN	$P_{\mathrm{cr}}/P_{\mathrm{cr}}^{\max}$ %
$f_{1}^{0}=1$, $P_{cr}^{0}=1$	28.9	93.4%	21.2	93.4%
$f_{1}^{0}$ and $P_{cr}^{0}$ obtained for $\Lambda ={\left[45/-45\right]}_{8}$	28.7	92.5%	21.1	92.8%
$f_{1}^{0}=f_{1}^{max}$, $P_{cr}^{0}=P_{cr}^{max}$	28.7	92.5%	21.1	92.9%

**Table 4:** Nadir points in SM approach.

Scaling Factors	$f_{1}$ Hz	$f_{1}/f_{1}^{\max}$ %	$P_{\mathrm{cr}}$ MN	$P_{\mathrm{cr}}/P_{\mathrm{cr}}^{\max}$ %
$f_{1}^{0}=1$, $P_{cr}^{0}=1$	29.1	94.0%	21.4	94.3%
$f_{1}^{0}$ and $P_{cr}^{0}$ obtained for $\Lambda ={\left[45/-45\right]}_{8}$	29.1	94.0%	21.3	93.9%
$f_{1}^{0}=f_{1}^{max}$, $P_{cr}^{0}=P_{cr}^{max}$	29.1	94.0%	21.4	94.0%

**Table 5:** Nadir points for different values of κ.

κ	$f_{1}$ Hz	$f_{1}/f_{1}^{\max}$ %	$P_{\mathrm{cr}}$ MN	$P_{\mathrm{cr}}/P_{\mathrm{cr}}^{\max}$ %
0.2	29.1	94.0%	21.3	93.9%
0.4	38.6	94.3%	39.4	93.7%
0.6	45.5	94.2%	59.9	93.8%
0.4	52.4	94.5%	87.4	94.3%

## Abbreviations

The following abbreviations are used in this manuscript:

GA	Genetic Algorithms
DNN	Deep Neural Networks
FE	Finite Elements
FEM	Finite Element Method
CL	Curriculum Learning
MOO	Multi-Objective Optimization
SM	Scalarization Method
PF	Pareto Front
NP	Nadir Point
